# Flubendazole carbonyl reduction in drug-susceptible and drug-resistant strains of the parasitic nematode *Haemonchus contortus*: changes during the life cycle and possible inhibition

**DOI:** 10.1186/s13567-023-01264-9

**Published:** 2024-01-15

**Authors:** Nikola Rychlá, Martina Navrátilová, Eliška Kohoutová, Lucie Raisová Stuchlíková, Karolína Štěrbová, Josef Krátký, Petra Matoušková, Barbora Szotáková, Lenka Skálová

**Affiliations:** Department of Biochemical Sciences, Faculty of Pharmacy, Charles University, Heyrovského, 1203 Hradec Králové, Czech Republic

**Keywords:** Anthelmintics, drug biotransformation, helminths, inhibitors, *Strongyloides*

## Abstract

Carbonyl-reducing enzymes (CREs) catalyse the reduction of carbonyl groups in many eobiotic and xenobiotic compounds in all organisms, including helminths. Previous studies have shown the important roles of CREs in the deactivation of several anthelmintic drugs (e.g., flubendazole and mebendazole) in adults infected with the parasitic nematode *Haemonchus contortus*, in which the activity of a CRE is increased in drug-resistant strains. The aim of the present study was to compare the abilities of nematodes of both a drug-susceptible strain (ISE) and a drug-resistant strain (IRE) to reduce the carbonyl group of flubendazole (FLU) in different developmental stages (eggs, L1/2 larvae, L3 larvae, and adults). In addition, the effects of selected CRE inhibitors (e.g., glycyrrhetinic acid, naringenin, silybin, luteolin, glyceraldehyde, and menadione) on the reduction of FLU were evaluated in vitro and ex vivo in *H. contortus* adults. The results showed that FLU was reduced by *H. contortus* in all developmental stages, with adult IRE females being the most metabolically active. Larvae (L1/2 and L3) and adult females of the IRE strain reduced FLU more effectively than those of the ISE strain. Data from the in vitro inhibition study (performed with cytosolic-like fractions of *H. contortus* adult homogenate) revealed that glycyrrhetinic acid, naringenin, mebendazole and menadione are effective inhibitors of FLU reduction. Ex vivo study data showed that menadione inhibited FLU reduction and also decreased the viability of *H. contortus* adults to a similar extent. Naringenin and mebendazole were not toxic at the concentrations tested, but they did not inhibit the reduction of FLU in adult worms ex vivo.

## Introduction

Parasitic nematodes (also called roundworms) represent a major burden in livestock and crop production [1, 2]. Diseases caused by parasitic nematodes are accompanied by various types of clinical complications with permanent and long-term effects on morbidity. Thus, the constant and regular control of these infections is vital for efficient and welfare-friendly livestock production. Veterinary pharmacotherapy with various anthelmintic drugs represents the basic strategy for the treatment and prophylaxis of nematodiases [2], although the effectiveness of the available anthelmintics has decreased due to increasing drug resistance in nematode populations [3, 4]. The development of various degrees of drug resistance among nematodes has been reported for all groups of anthelmintics, and the prevalence of resistance has greatly increased worldwide [3].

Drug resistance can be classified into two main categories: target-site resistance (TSR) and non-target-site resistance (NTSR). TSR is caused primarily by DNA mutation, which triggers an alteration in the structure of a target site, while NTSR is caused mainly by enhanced detoxification and elimination via the increased expression and activity of drug-metabolizing enzymes (DMEs) or by transporter upregulation [5, 6]. These proteins protect all organisms from the potential negative effects of drugs and other xenobiotics via deactivation and elimination. Nematodes possess a relatively large number of DME genes, many more than other classes of helminths [7]. As a result, nematodes are able to metabolize numerous anthelmintics effectively to form various types of anthelmintic metabolites [8, 9]. Based on these initial findings, the role of DMEs in drug resistance in nematodes has been studied intensively.

Flubendazole (FLU; [5-(4-fluorobenzoyl)-1*H*-benzimidazol-2-yl]-carbamic acid methyl ester), is a benzimidazole-type anthelmintic drug approved that was in 1980 for helminthiasis treatment in both veterinary and human medicine. FLU metabolism has been examined in several mammalian species, e.g., rats, pigs, cattle and sheep [10, 11]. In the liver, FLU is metabolized via carbonyl reduction, generating reduced FLU (FLU-R), as well as via hydrolysis to produce decarbamoylated FLU. Both of these FLU metabolites have shown significantly lower anthelmintic activity than the parent drug [12].

The nematode *Haemonchus contortus* (family *Trichostrongylidae*), a gastrointestinal parasite in ruminants, is able to reduce FLU, with FLU-R being the main metabolite formed in *H. contortus* ex vivo and in vitro [13]. In addition to reducing FLU, *H. contortus* also hydrolyses FLU and conjugates it with UDP-glucose [14]. In drug-resistant strains of *H. contortus*, significantly more FLU metabolites were formed ex vivo than those formed in the susceptible strain. In vitro data also revealed significantly increased activities of several carbonyl-reducing enzymes towards model substrates of resistant *H. contortus* strains [13, 14]. The reduction of carbonyl groups in xenobiotics (as well as many eobiotics) is generally catalysed by members of the short-chain dehydrogenase/reductase (SDR) and aldo/keto reductase (AKR) superfamilies. The genome of *H. contortus* contains 70 SDR genes and 24 AKR genes [7]. A comparison of SDR expression between the drug-susceptible and drug-resistant strains of *H. contortus* revealed that several SDRs had increased expression in the resistant strains [15]. In view of these findings, it may be assumed that increases in the expression and activity of carbonyl-reducing enzymes (CRE) might protect *H. contortus* from reactive carbonyl-bearing xenobiotics, including FLU. Until now, FLU reduction has been studied only in *H. contortus* adults [13, 14, 23, 24], with no information yet available regarding the possibility that lower developmental stages can deactivate FLU via carbonyl reduction. Although these drugs target the infectious and parasitic stages, eggs and free-living larvae can also encounter FLU in the environment that has been excreted from treated animals; thus, the eggs and free-living larvae have developed protective mechanisms against FLU toxicity.

Therefore, the present study was designed to investigate the ability of *H. contortus* during different developmental stages (eggs, larvae, and adults) to reduce FLU. The proportions of FLU-R formed in all developmental stages from drug-susceptible (ISE) and drug-resistant (IRE) strains of *H. contortus* were compared. As inhibiting FLU reduction may increase the anthelmintic efficacy of FLU, the effects of selected carbonyl-reducing enzyme inhibitor candidates on the reduction of FLU in *H. contortus* were evaluated in vitro on subcellular adult homogenate fractions and ex vivo on isolated adults cultivated in media.

## Materials and methods

### Chemicals

Flubendazole (FLU), the deuterated standard FLU-D_3_, NADPH, glycyrrhetinic acid, mebendazole, menadione, naringenin and silybin were purchased from Sigma Aldrich (Prague, Czech Republic). Luteolin was obtained from TRC Canada (Toronto, Canada), and glyceraldehyde was purchased from VWR Chemicals BHD (England). Dimethyl sulfoxide (DMSO), formic acid (FA) (LC‒MS LiChropur™, 97.5–98.5%) and acetonitrile (ACN) (UHPLC‒MS grade) were obtained from VWR International s.r.o. (Prague, Czech Republic). Ultrapure water was prepared from deionized water using a Milli-Q ultrapure water purification system (Millipore, Bedford, MA, USA). Liquid sterile-filtered RPMI-1640 medium was purchased from Biosera (Biotech, Prague, Czech Republic). An ATP Bioluminescence Assay Kit CLS II was purchased from Roche (Mannheim, Germany).

### Sheep breeding and infection

In this study, two *H. contortus* strains were used: an inbred susceptible Edinburgh strain (ISE, MHco3) and an inbred resistant Edinburgh strain (IRE, MHco5) [16]. The sheep (as the host of *H. contortus*) were bred and slaughtered in accordance with Czech slaughtering rules for farm animals and the Protection of Animals from Cruelty Act No. 246/1992, Czech Republic. The experimental protocol was evaluated and approved by the Ethics Committee of the Ministry of Education, Youth and Sports (MSMT-25908/2019).

Four parasite-free lambs (6–9 months old) were orally infected with 6000 third-stage larvae (L3) of the ISE (two lambs) or IRE (two lambs) strain of *H. contortus*. The required amount of L3 was suspended in 10 mL of water and administered to the sheep by gavage.

### Isolation of *H. contortus* eggs and larvae

Five weeks after infection, sheep faeces were collected in plastic bags for 12 h, mixed and homogenized in cold tap water. The nematode eggs were isolated at room temperature using three sieves with different mesh sizes (250 μm, 100 μm and 25 μm). The content from the third sieve (25 μm) was collected in 50 mL falcon tubes and centrifuged at 1600 × *g* for 3 min. The supernatant was removed, and the volume was adjusted by adding Sheather’s flotation sucrose solution (1.27 g cm^−3^), and the sample was mixed and centrifuged at 1000 × *g* for 3 min. The eggs were washed with tap water and centrifuged at 1600 × *g* for 3 min. This last step was repeated until the desired egg purity, which was determined microscopically, was achieved; after this, the eggs were counted and kept overnight at 4 °C. L1/2 larvae were obtained from the isolated eggs, which were incubated in tap water for 24 h at 27 °C on a sieve with a mesh size of 20 μm, and the hatched L1/2 larvae were collected from under the sieve. L3 larvae were produced from the eggs by incubating humidified faeces from the infected lambs at 27 °C for 1 week, after which the L3 larvae were collected, filtered through a 20 μm sieve over 4 h and stored in tap water at 4 °C until incubation, after which the fitness of the larvae was checked microscopically.

### Isolation of adult nematodes

Seven weeks after infection, the lambs were stunned and exsanguinated. The agar method was used to isolate the adult worms from the sheep abomasum, as described previously [17, 18]. The sex of the adult worms was determined according to morphological characteristics, and males and females were separated manually. For the preparation of subcellular fractions, adult worms were stored at −80 °C.

### Preparation of subcellular fractions from adult nematodes

The subcellular fractions from the adult nematodes were prepared by differential ultracentrifugation of the homogenate. One gram of frozen nematodes (ISE or IRE strain) was thawed at room temperature, mixed with 6 mL of 0.1 M phosphate-buffered saline (PBS, pH 7.4) and homogenized with a Potter-Elvehjem tissue homogenizer. The homogenate was centrifuged at 20 000 × *g* for 60 min at 4 °C, and subsequently, the supernatant was ultracentrifuged at 105 000 × *g* for 65 min at 4 °C. The obtained final supernatant (cytosolic-like fraction) was aliquoted and stored at −80 °C [19]. The protein content in each subcellular fraction was assayed using the bicinchoninic acid (BCA) method according to the manufacturer’s protocol (Sigma-Aldrich, Prague, Czech Republic).

### Evaluation of the toxicities of inhibitor candidates to *H. contortus* adults ex vivo

The effect of the selected inhibitors on the viability of ISE *H. contortus* adults was evaluated using an ATP content assay as previously described [20]. Briefly, adult *H. contortus* nematodes (eight males and four females) were incubated in 1 mL of RPMI-1640 medium supplemented with 0.8% glucose, 0.25 µg/mL amphotericin B, 10 U/mL penicillin, 10 µg/mL streptomycin and 10 mM HEPES at 37 °C with 5% CO_2_ for 48 h with or without inhibitor at a concentration of 100 µM (20 µM for mebendazole) that had been predissolved in DMSO (the final DMSO concentration in was 0.1% v/v). Medium supplemented with pure DMSO (0.1%) was used as a negative control, and medium supplemented with 5 µM levamisole (LEV) was used as a positive control. The samples were prepared with 4 replicates. After 48 h of incubation, the adult nematodes were collected, washed 3 times with PBS, and stored in 100 µL of freshly prepared sonification solution (SONOP, 70% v/v, with 2 mM EDTA, pH 10.9) at −80 °C. Prior to analysis, the samples were homogenized using a FastPrep-24 5G homogenizer (MP Biomedicals, France) with 1–2 mm ceramic beads for 30 s (6 m/s) in Tris/EDTA buffer (100 mM Tris, 2 mM EDTA, pH 8.0). After homogenization, the samples were centrifuged at 13 200 × *g* for 10 min at 4 °C. The ATP concentration was determined with an ATP Bioluminescence Assay Kit CLS II (Roche, Mannheim, Germany) according to the manufacturer’s protocol. Subsequently, the ATP concentration was normalized to the total amount of protein in the sample. The protein content was measured using the bicinchoninic acid method. Modifications from the manufacturer’s protocol are detailed in [21].

### Ex vivo study of FLU metabolism by *H. contortus* in different life stages

Eggs and larvae were incubated in 24-well plates (TPP® tissue culture plates, Sigma Aldrich, Czech Republic), with each well containing approximately 60 000 eggs, L1/2 or L3 larvae suspended in 2 mL of tap unsterilized water. FLU (predissolved in DMSO) was added to the samples to produce final concentrations of 0.5, 1 and 5 µM FLU. The concentration of DMSO in the samples and control was 0.15%. The mixtures were incubated for 24 h at 27 °C. Adult nematodes were incubated ex vivo in RPMI-1640 medium enriched with 0.8% glucose, 0.25 µg/mL amphotericin B, 10 U/mL penicillin, 10 µg/mL streptomycin, and 10 mM HEPES, as described previously [22]. Fifteen males or ten females (to obtain approximately the same final sample weight) were placed into one well of the well plate with 2 mL of RPMI medium containing FLU at the final concentrations noted above. In the ex vivo inhibition study, the inhibitors (predissolved in DMSO) at a concentration of 100 µM (or 20 µM for mebendazole) were added to the sample together with FLU. The concentration of DMSO in the samples was 0.15%. The 24-well plates with adults were incubated for 24 h at 37 °C in a CO_2_ incubator (5% CO_2_, D180-P CO_2_ Incubator, RWD Life Science, Inc., USA). The samples were prepared in 4–6 replicates. Biological blank samples containing pure DMSO instead of FLU in DMSO solution as well as chemical blank samples without biological material were prepared and incubated in the same way as the samples described above. The eggs, larvae and adults were washed three times with PBS and stored at −20 °C until LC‒MS/MS analysis.

### In vitro inhibition study of FLU reduction in the subcellular fractions from adults

The reduction of FLU and the effects of potential inhibitors were studied in cytosolic-like fractions of *H. contortus* homogenates (from the ISE and IRE strains). The total volume of the reaction mixture was 100 µL, and each sample contained 50 µL of the cytosol-like fraction (protein content 0.3–0.5 mg/mL), FLU as a substrate (10 µM) predissolved in DMSO, NADPH (1 mM), inhibitor (10 or 100 µM; 10 or 20 µM for mebendazole) predissolved in DMSO, and 0.1 M Na-phosphate buffer, pH 7. The samples were prepared with 6 technical replicates in 3 independent experiments. The reactions were carried out at 37 °C for 4 h under aerobic conditions to produce enough FLU-R for detection. After 4 h, the reaction was stopped with the addition of 300 µL of ice-cold 100% ACN and centrifuged at 15 000 × *g* for 15 min at 4 °C. The supernatant was removed, and after the addition of FLU-D_3_ as an internal standard (IS), the solvent was evaporated using a centrifuge concentrator (Concentrator Plus, Eppendorf, Germany) set on vacuum-alcoholic mode at 30 °C for 5 h and the residue was stored (−20 °C).

### Sample preparation for LC‒MS/MS analysis

The eggs, larvae, and adult *H. contortus* nematodes (females and males separately) were resuspended in cooled 0.1 M Na-phosphate buffer (pH 7.4) supplemented with 0.1 µM IS and homogenized six times in 30 s cycles at 6 m/s using a FastPrep-24 5G homogenizer with ceramic beads (size 1.4–1.0 mm). After homogenization, the samples were centrifuged at 3000 × *g* for 5 min. The protein concentration was determined using a bicinchoninic acid assay according to the Sigma-Aldrich protocol.

Solid-phase extraction was performed using SPE Strata-X columns with a Visiprep SPE vacuum manifold apparatus (Supelco®, 12 ports, PA, USA). The columns were activated with 1 mL of 100% ACN (LC‒MS grade) and washed with 1 mL of H_2_O. The next step involved loading 1 mL of sample (eggs, L1/2 larvae or adult nematode homogenate with IS) and washing with 2 mL of 10% ACN in 90% ultrapure water. Elution was performed with 1 mL of 100% ACN, and the eluate was collected in glass vials (1.5 mL) suitable for HPLC‒MS/MS analysis. The solvent was evaporated using a centrifuge concentrator (Concentrator Plus) set in vacuum-alcoholic mode at 30 °C for 5 h and the residue was stored (−20 °C) until LC–MS/MS analysis.

Prior to analysis, the samples were reconstituted in 30 µL of ACN and subsequently mixed for 5 min using a tube roller (Mx-T6-S, Dlab, CA, USA), followed by an additional 5 min of sonication in a bath. Then, 70 µL of ultrapure water was added to the samples before the tube rolling and sonication steps were repeated. The resulting extracts were then passed through disposable PTFE syringe filters with a pore size of 0.22 μm (Labicom, Czech Republic), transferred to glass vials with glass inserts (Agilent Technologies, Waldbronn, Germany) and analysed immediately after reconstitution.

### UHPLC‒MS/MS analysis

A Nexera X2 UHPLC system, which consisted of a binary pump, vacuum degasser, temperature-controlled autosampler (set at 15 °C) and temperature-controlled column compartment (set at 40 °C), was utilized for the separation process (Shimadzu, Kyoto, Japan).

The analytes were separated using a Zorbax Bonus RP C18 column (2.1 mm × 150 mm, 1.8 μm particle size) with a matching guard column (Agilent Technologies, Waldbronn, Germany) at a flow rate of 0.4 mL/min. The injection volume for all the analyses was 1 µL. Separation of the samples was achieved through linear gradient elution comprising solutions A (dH_2_O + 0.1% FA) and B (ACN + 0.1% FA). The gradient protocol was as follows: initial treatment with 20% B for 1 min, followed by a linear increase to 95% B over 3 min. These conditions were maintained for 2 min, followed by a switch back to the starting condition of 20% B and 5 min of re-equilibration. The total run time was 10 min.

MS/MS analysis was conducted using a triple quadrupole mass spectrometer (LCMS-8030; Shimadzu, Kyoto, Japan) with an ESI ionization source operating in positive SRM mode (Table 1). The ESI parameters were analysed for the analytes according to the design of the experiments, which allowed us to find the best ionization conditions for the individual compounds. The ionization source was configured with the following parameters: capillary voltage, 0.5 kV for FLU-R and 1.5 kV for both FLU and FLU-D_3_; heat block temperature, 400 °C; DL line, 250 °C; nebulizing gas flow rate, 2.5 L/min; and drying gas flow rate, 12 L/min. Nitrogen was used as both the nebulizing and drying gas, while argon served as the collision gas (pressure 270 kPa) for the MS/MS experiments. Data acquisition and processing were performed using LabSolution LCMS software version 5.109 (Shimadzu, Kyoto, Japan).

**Table 1:** **List of analytes and their characteristics [retention time, (t**_**R**_**), precursor ion and production ions with collision energy]**

Compound name	Molecular formula	t_R_ [min]	Precursor ion [M+H]^+^, *m/z*	Product ion [M+H]^+^, *m/z* (collision energy [eV])
FLU	C_16_H_12_FN_3_O_3_	3.82	314.10	281.80 (−22); 122.95 (−39); 94.95 (−50)
FLU-D_3_	C_16_D_3_H_9_FN_3_O_3_	3.82	317.10	281.90 (−24); 122.85 (−40); 94.95 (−53)
FLU-R	C_16_H_14_FN_3_O_3_	2.11	316.10	283.95 (−26); 97.00 (−46); 124.90 (−38)

### Statistical analysis

All statistical analyses were performed using GraphPad Prism software 9.5.1 (GraphPad Prism, USA), with differences considered significant at *P* < 0.05. The reported data are expressed as the mean ± SD of 6 technical replicates for metabolic analysis and 3 technical replicates for in vitro reactions using cytosolic-like fractions prepared in a pool (due to the critically low amount of biological material) from 3 independent experiments. The FLU-R concentrations were statistically compared using the Mann‒Whitney test and one-way ANOVA with a post hoc Holm‒Sidak multiple comparison test. Likewise, statistical comparisons of the in vitro data were performed using one-way ANOVA with a post hoc Holm‒Sidak test.

## Results

### Biotransformation of FLU ex vivo with *H. contortus* in different life stages

The eggs, L1/2 and L3 larvae, and adult *H. contortus* nematodes (ISE and IRE strains) were incubated with FLU. After 24 h of incubation, the amount of the major metabolite FLU-R was semiquantified using LC‒MS. The amounts of the metabolites were quantified by calculating the ratio of the metabolite peak area to the peak area of the internal standard, and these values were normalized to milligrams of protein mass in the eggs, larvae (L1/2 and L3) or adult nematodes during incubation.

**Figure 1:**
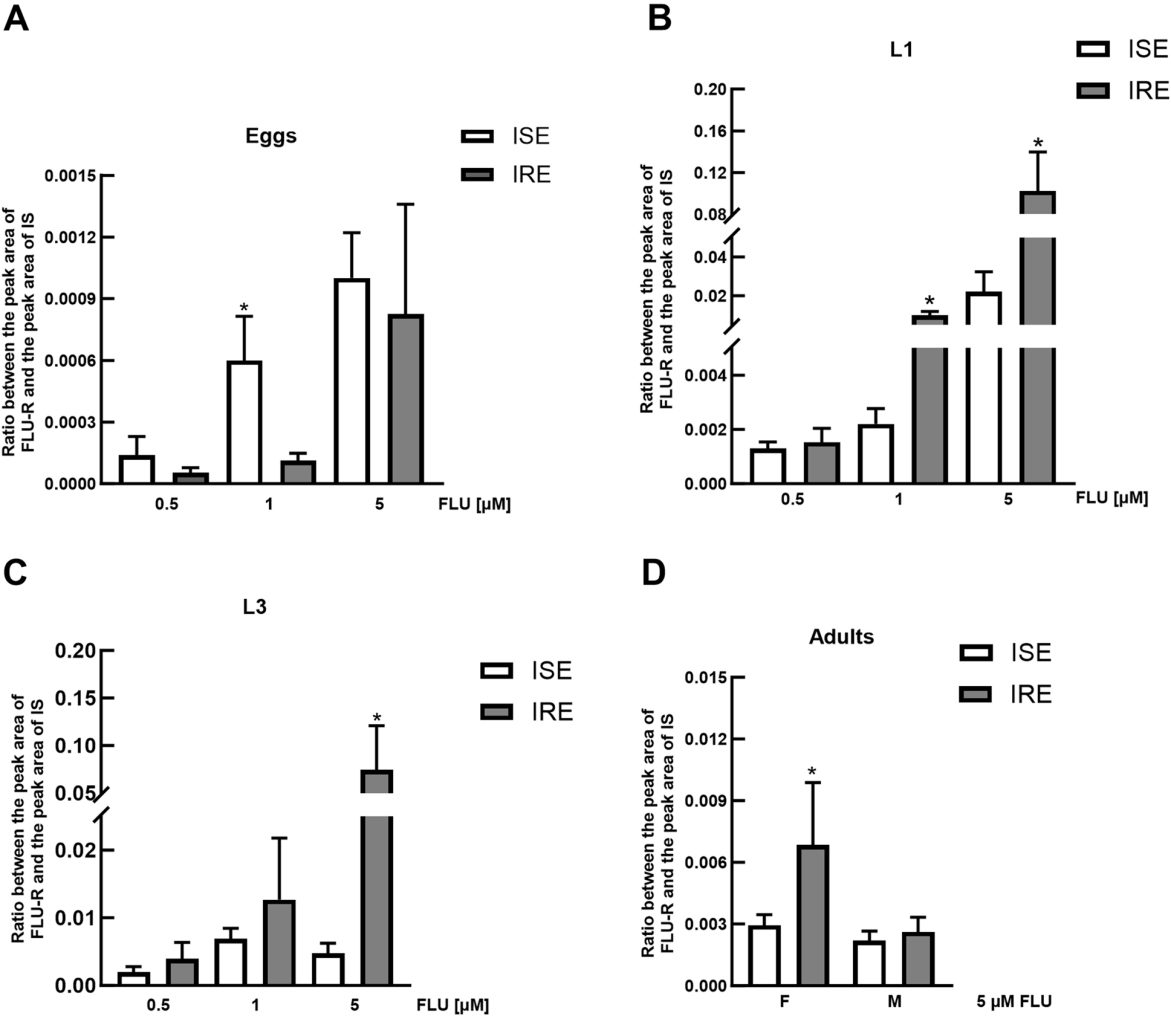
**Formation of FLU-R in** ***Haemonchus contortus*** **at various developmental stages.** Quantities of FLU-R found in homogenates after 24 h of ex vivo incubation of eggs (**A**), L1/2 (**B**) and L3 (**C**) larvae (with 0.5 µM, 1 µM, and 5 µM FLU), and adults (**D**) (with 5 µM FLU) from two *H. contortus* strains (ISE, a drug-susceptible strain, and IRE, a drug-resistant strain). *Indicates a significant difference between the ISE and IRE strains within the drug concentration range at *P* < 0.05.

The results obtained from the eggs and larvae incubated with 0.5 µM, 1 µM and 5 µM FLU showed concentration-dependent FLU-R formation (Figures 1A–C). The results also revealed that the lowest carbonyl reduction activity occurred in the eggs, the activity in the larvae was higher, and the highest activity occurred in the adults. In addition, a sex difference in FLU reduction was detected, especially in the IRE strain, with females exhibiting greater CRE activity. A comparison of the data from the ISE and IRE strains revealed that the eggs of the IRE strain did not form more FLU-R, while the larvae of the IRE strain were able to reduce FLU more extensively than the ISE strain; this difference was significant at 1 and 5 µM FLU. Enhanced FLU-R production was also found in the adult females of the IRE strain in comparison to those of the ISE strain (Figure 1D).

### In vitro inhibition study

The effects of selected carbonyl-reducing enzyme inhibitors on the reduction of FLU were studied in cytosolic-like fractions prepared from *H. contortus* adults from the ISE and IRE strains. The following inhibitor candidates were used: glycyrrhetinic acid, naringenin, silybin, luteolin, glyceraldehyde, and menadione, each at concentrations of 10 and 100 µM, and mebendazole at concentrations of 10 and 20 µM (due to its low solubility).

**Figure 2:**
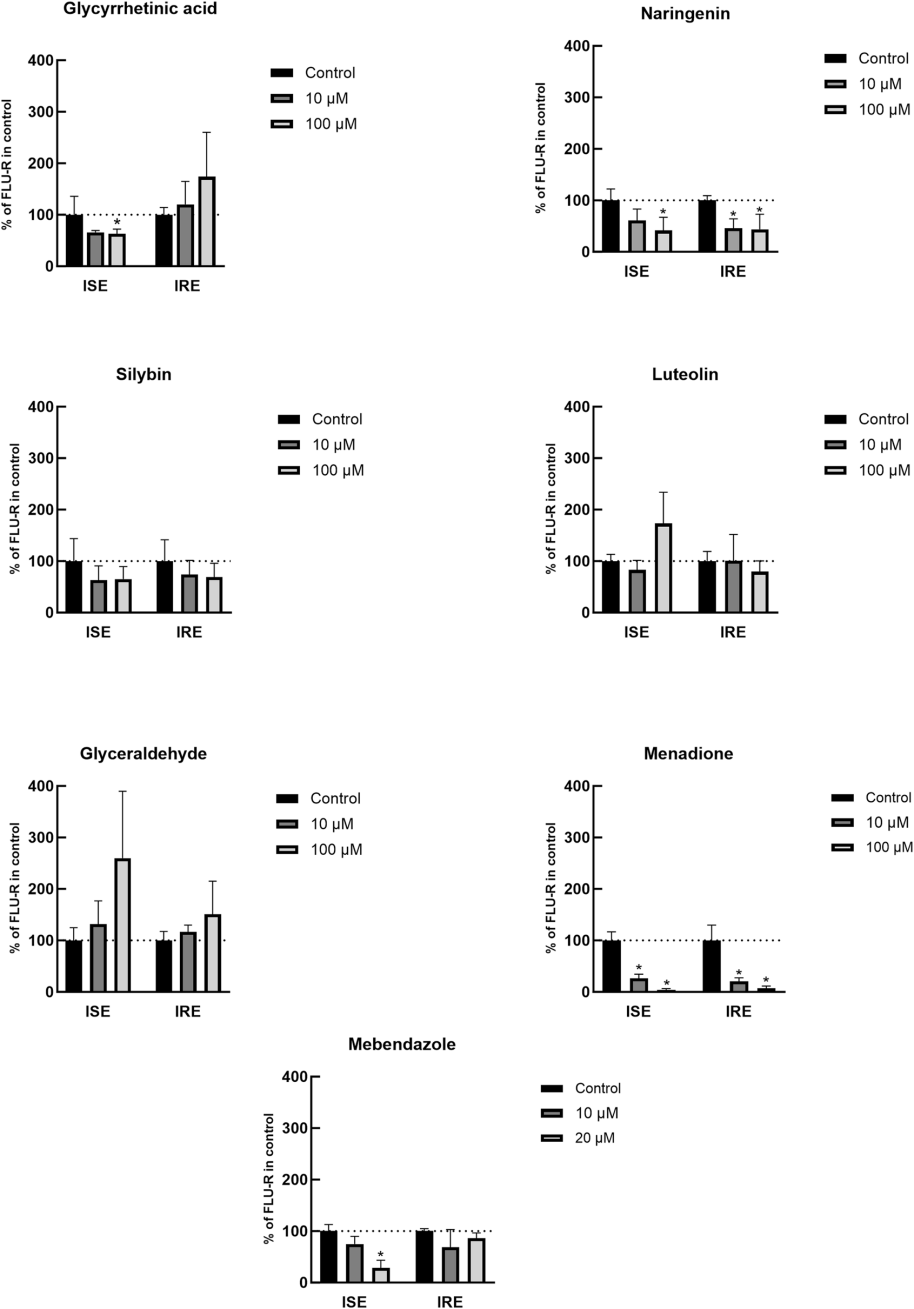
**Effects of possible inhibitors on FLU-R formation in vitro.** The production of FLU-R in the cytosol-like fractions from *H. contortus* adults (ISE, a drug-susceptible strain, and IRE, a drug-resistant strain) inhibited by chosen inhibitors relative to that produced in the inhibitor-free control, which represents 100% formed FLU-R. Data are presented as the mean ± SD. *Indicates a significant difference between the control and inhibitor groups within the strain (ISE or IRE) at *P* < 0.05.

The results (Figure 2) revealed that several compounds significantly inhibited the reduction of FLU within the cytosolic-like fraction of *H. contortus* adults. Specifically, glycyrrhetinic acid (100 µM) inhibited FLU reduction in the ISE strain by 37.3%; naringenin (100 µM) by 58.5% in the ISE strain and by 56.3% in the IRE strain, menadione (100 µM) by 96.1% in the ISE strain and by 93% in the IRE strain; and mebendazole (20 µM) by 71.2% in the ISE strain. Among these inhibitors, menadione has been proven to be the most effective inhibitor of FLU reduction. On the other hand, silybin, luteolin and glyceraldehyde did not significantly inhibit FLU-R formation in *H. contortus* in vitro.

A comparison of the cytosolic-like fractions from adults of the ISE and IRE strains revealed that naringenin and menadione similarly inhibited the reduction of FLU by both strains. However, glycyrrhetinic acid and mebendazole were found to have inhibitory effects only with the ISE strain, with no significant impact on FLU reduction by the IRE strain.

### Toxicity test

**Figure 3:**
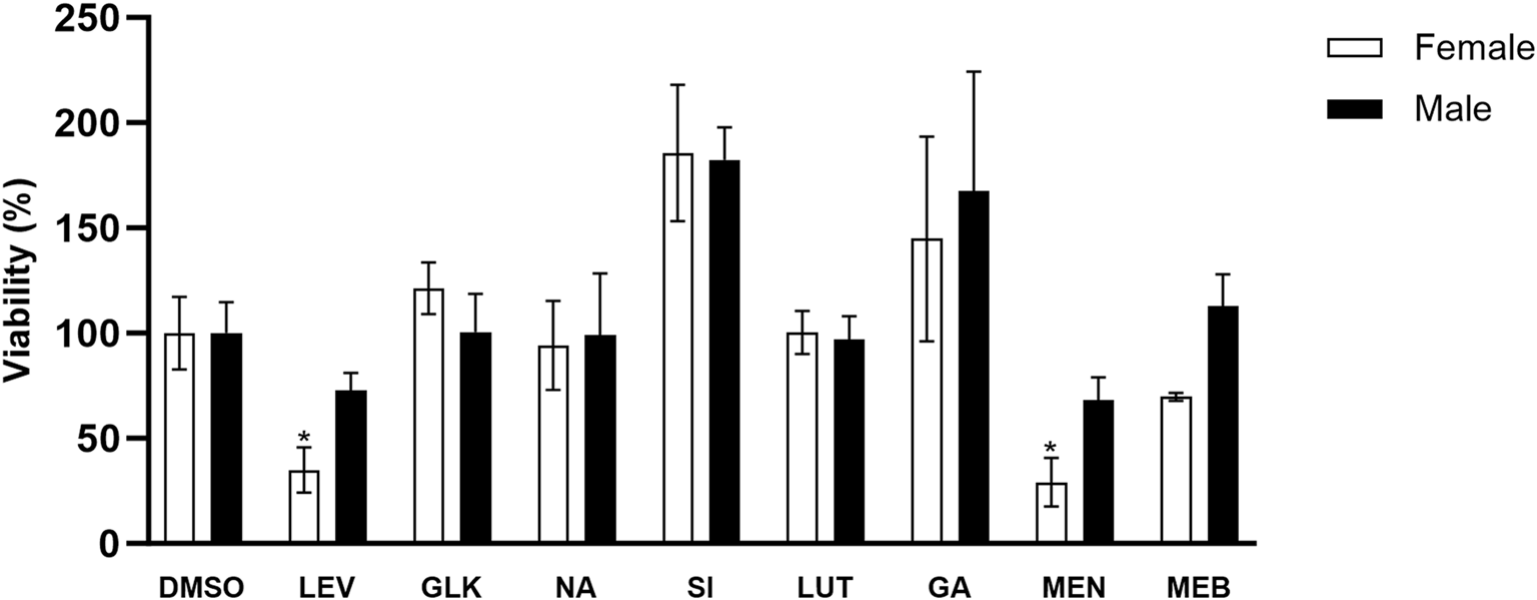
**Effects of selected inhibitors on the ex vivo viability of adult drug-susceptible** ***H. contortus*** **(strain ISE) determined by ATP content.** The presented data are expressed as a percentage of the control (100%) and were normalized to the protein concentration in the sample (mean ± SD). *Indicates a significant difference between the control and inhibitor samples at *P* < 0.05. F: female; M: male; DMSO: dimethylsulfoxide (0.1%); LEV: levamisole (5 µM; typical IC_50_ in the ISE strain); GLK: glycyrrhetinic acid (100 µM); NA: naringenin (100 µM); SI: silybin (100 µM); LUT: luteolin (100 µM); GA: glyceraldehyde (100 µM); MEN: menadione (100 µM); MEB: mebendazole (20 µM).

The effects of selected inhibitors and levamisole (used as a positive control) on *H. contortus* viability were evaluated in adult nematodes ex vivo. The results (Figure 3) showed the significant toxicity of menadione (and levamisole) to nematodes. The other inhibitors tested did not significantly decrease the viability of the nematodes at the concentrations used. On the other hand, silybin increased the viability of both female and male nematodes.

### Ex vivo inhibition study

**Figure 4:**
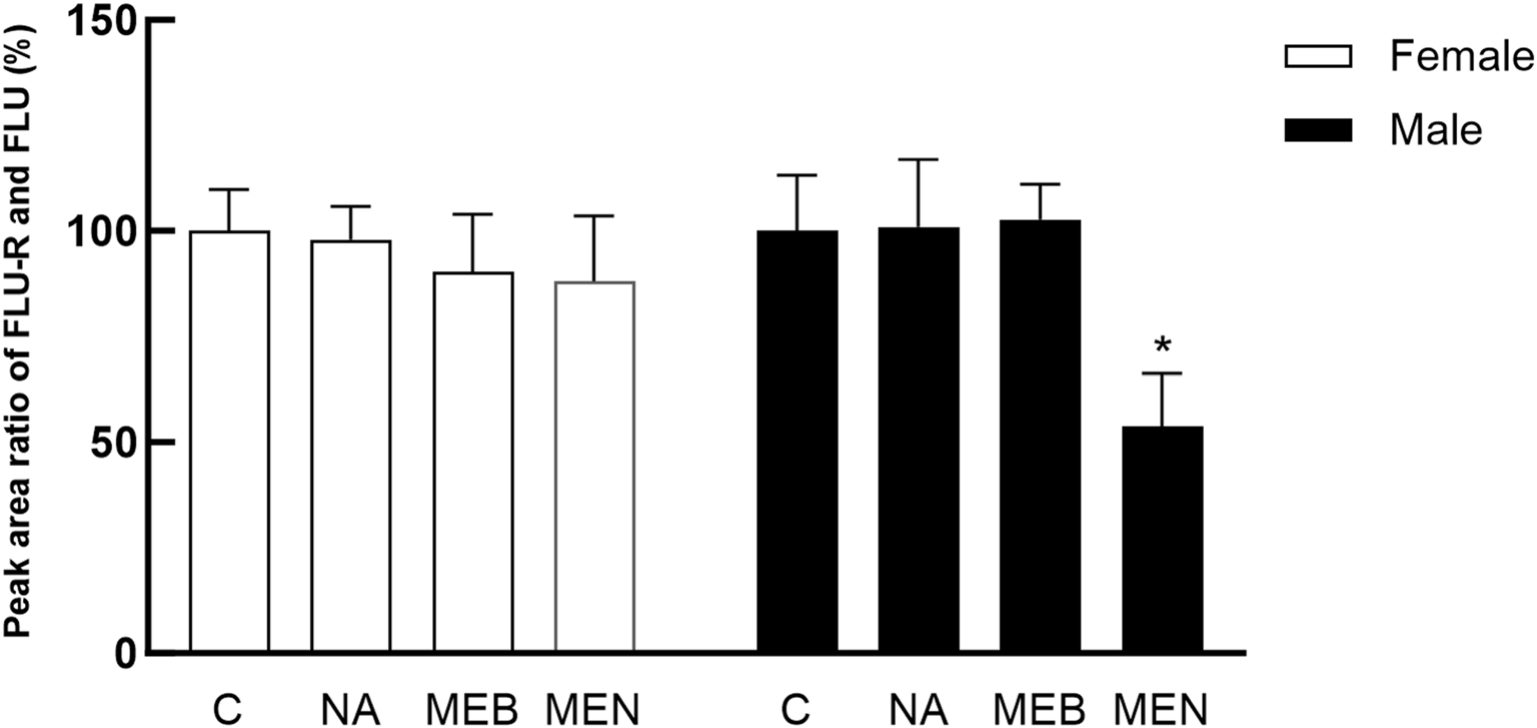
**Inhibitory effects of naringenin (NA, 100 µM), menadione (MEN, 100 µM), and mebendazole (MEB, 20 µM) on the production of FLU-R by** ***H. contortus*** **adults of the ISE strain ex vivo.** The data shown are expressed as the ratio between the peak area of FLU-R and the peak area of FLU (%). The control reaction (C) without inhibitor represented 100% of the formed product. The data are expressed as the mean ± SD.

With the goal of determining the inhibition of FLU-R formation in *H. contortus* ex vivo, adult females and males (ISE strain) were incubated with 5 µM FLU with or without three promising inhibitors, naringenin (100 µM), menadione (100 µM), and mebendazole (20 µM), for 24 h. The obtained results are presented in Figure 4. Only menadione significantly decreased FLU-R formation in *H. contortus* males ex vivo.

## Discussion

All organisms protect themselves from the potential toxicity of xenobiotics through biotransformation. While the biotransformation pathways of most xenobiotics and the corresponding enzymes that catalyse these reactions are well known in humans and experimental animals, information about many the reactions between xenobiotics and enzymes in other organisms, e.g., parasitic nematodes, is insufficient. Moreover, much more attention has been devoted to oxidizing enzymes such as cytochrome P450s and conjugating enzymes (e.g., glutathione *S*-transferases and UDP-glycosyltransferases) than to xenobiotic-reducing enzymes [7]. Although reduction pathways are generally employed less often than oxidation pathways in aerobic organisms, the former predominate for some types of compounds, e.g., carbonyl compounds, which are transformed into less reactive alcohols through reduction of the carbonyl group [23]. This is also true for FLU, a carbonyl-bearing anthelmintic. The deactivation product FLU-R [12] is the only FLU metabolite that forms in the human liver [24], and it is the main metabolite in rats, pigs, cattle and sheep [10, 11]. The nematode *H. contortus* can also reduce FLU, with FLU-R being the main metabolite formed in adult nematodes ex vivo and in vitro [13]. Importantly, adult nematodes of drug-resistant strains have been found to reduce FLU more effectively than those of drug-susceptible strains [13, 25, 26]. Nevertheless, since FLU reduction has thus far been studied only in *H. contortus* adults, there is a need to determine whether lower developmental stages can also deactivate FLU via carbonyl reduction and whether FLU-R production is increased in drug-resistant strains compared with drug-sensitive strains.

In the present study, the eggs, L1/2 and L3 larvae, adult females, and adult males of *H. contortus* were treated with FLU, and after 24 h of incubation, the amount of FLU-R produced was quantified. Two strains, a drug-susceptible strain (ISE) and a drug-resistant strain (IRE), were compared. Although the results obtained show for the first time that all developmental stages of *H. contortus* can deactivate FLU, only very small amounts of FLU-R were detected. As previously reported, the expression of reductases differs between the stages. Therefore, we believe that the reductase(s) responsible for FLU reduction may be expressed mainly at higher stages; for example, *sdr5*, which has the lowest expression in eggs [15]. The low permeability of some compounds through egg cuticles can result in the underestimations of metabolic rate, although this is unlikely to be the case with FLU, as metabolites of the structurally similar benzimidazole, albendazole (both sulfone and sulfoxide forms), have been found to accumulate in egg homogenates in greater amounts than in other developmental stages [17]. The larvae reduced FLU much more effectively than the eggs did, although the highest production of FLU-R was observed in the adults, specifically in IRE females. The differences between the strains were most pronounced in the larvae, in which FLU-R formation was 7 times greater in the IRE strain than in the ISE strain. On the other hand, while higher FLU-R production was observed in ISE eggs than in IRE eggs, the extent of FLU deactivation in eggs was so low that it could not represent an effective defence mechanism. Quantitative PCR analysis of the expression of short-chain dehydrogenases across the life cycle of *H. contortus* revealed high expression of several genes in larvae and adults. Furthermore, some genes exhibited increased expression in resistant larvae, although only *sdr1* exhibited increased expression in IRE females compared with ISE females [15]. Therefore, the enzyme SDR1 is currently a strong candidate that could be responsible for FLU reduction in *H. contortus*. Nevertheless, verification via recombinant enzyme expression or RNAi will be necessary. In addition, the ability of FLU to induce SDR expression has yet to be investigated. The difference in SDR expression between strains has been assessed only at the constitutive level, with the potential of inducibility by FLU remaining unexamined thus far.

As FLU reduction leads to FLU deactivation [12], inhibiting this reaction might increase FLU efficacy, particularly in drug-resistant strains with increased FLU reduction capabilities [13, 14]. Several possible inhibitors that may limit FLU reduction in *H. contortus* adults were tested. Natural compounds with proven inhibitory effects on certain carbonyl-reducing enzymes and low toxicity to mammals were selected. Menadione, also known as vitamin K3, is a well-known substrate and competitive inhibitor of human carbonyl reductase 1 [14, 27]. Menadione has also been shown to be a substrate of carbonyl reductase in *Daphnia* spp. [28]. The flavonoids luteolin and naringenin inhibit human CBR1, with luteolin acting as a potent CBR1 inhibitor [29, 30]. The flavonolignan silybin has been described as an inhibitor of aldehyde oxidase and xanthine oxidase in rats [31]. Glyceraldehyde is a substrate (and competitive inhibitor) of human aldose reductase (AKR1B1) [32]. Glycyrrhetinic acid, a biologically active triterpenoid acid, inhibits several hydroxysteroid dehydrogenases, e.g., 11-β-HSD1 and AKR1C1-3 [33, 34]. In addition to these natural compounds, the effect of mebendazole, a structurally similar anthelmintic, on FLU reduction was also evaluated.

The results of the in vitro inhibition study showed pronounced concentration-dependent inhibitory effects of naringenin and menadione on the reduction of FLU. Since the extent of inhibition was nearly the same in the ISE and IRE strains, it can be assumed that this FLU-reducing enzyme is similarly expressed in both strains. Interestingly, luteolin did not inhibit FLU-R formation, although this flavone is a stronger inhibitor of human CBR1 than the flavanone naringenin [29] and CBR1 was identified as the main enzyme involved in reducing FLU in the human liver [35]. There are 46 members of the short-chain dehydrogenase family in *H. contortus*; however, the main FLU-reducing enzyme has yet to be identified. Nevertheless, due to the considerable structural divergence between human CBR1 and the CBR-like enzymes from *H. contortus* (e.g., *Hco*_SDR4, the most similar enzyme, shares only 35.4% similarity), we cannot rule out differences in the extent of inhibition. Glycyrrhetinic acid and mebendazole inhibited FLU reduction only in the ISE strain. This difference might indicate the redundancy of certain carbonyl-reducing enzymes in the IRE strain that can replace the inhibited enzyme(s) to reduce FLU. These findings agree with the results of a previous study that demonstrated the increased expression of several members of the SDR superfamily in the IRE strain compared to the ISE strain [15]. Nonetheless, it is important to note that glycyrrhetinic acid has been shown to inhibit enzymes from the AKR1C subfamily in humans [34]; thus, participation from some enzymes of the AKR superfamily in reducing FLU in *H. contortus* is also possible. Although the *H. contortus* genome contains 24 AKR genes [7], no information on their substrate specificities or differences in AKR gene expression among *H. contortus* strains has been reported.

As the ex vivo study was being performed, the toxicities of potential FLU-reductase inhibitors were also tested in *H. contortus* adults. Females and males were treated separately to observe possible sex-specific differences in the effects of the inhibitors. At the highest concentrations tested, while the majority of the tested inhibitors did not significantly decrease the viability of the nematodes, menadione did have significant toxicity. Moreover, silybin caused increased the viability of both males and females, which was not surprising, as silybin displays protective effects in various systems [36]. Surprisingly, neither the known anthelmintic mebendazole nor the levamisole positive control decreased the viability of the males. Nonetheless, this result could be an artifact of the ATP assay, as under certain stress conditions, the level of ATP has been shown to increase.

In contrast, the inhibitory effects of naringenin and mebendazole, the most potent in vitro inhibitors that were nontoxic ex vivo, were not confirmed in the isolated adult nematodes ex vivo since neither compound was able to inhibit FLU reduction under the conditions tested. The discrepancy between the results of the in vitro and ex vivo studies might have been caused by differences in the accessibility of FLU to the reducing enzymes and inhibitory agents when the enzymes and compounds are homogenous in the cytosolic-like fraction and when the compartmentalizing barriers have been removed. This situation contrasts that of the ex vivo system, where a cuticle with low permeability, spatially and substrate-selective gut sorption, and organelle-facilitated detoxification and export from tissues can cause localized differences in the location and concentration of both enzymes and compounds. As FLU is highly lipophilic, it is readily able to passively diffuse into and throughout nematodes. On the other hand, hydrophilic compounds such as naringenin need to enter by other means (if they are able to do so at all); therefore, sufficient concentrations of the hydrophilic agents may not be in the same location as the reducing enzymes to inhibit FLU reduction. Unfortunately, due to the limited number of worms, we could not determine the internal concentrations of all the inhibitors, and due to a lack of recombinant enzymes from *H. contortus*, we cannot quantify the inhibition of the specific enzymes involved. It was slightly surprising that mebendazole, which is also lipophilic (although less so than flubendazole), did not competitively inhibit FLU reduction, although FLU may be a better substrate than mebendazole for the responsible reductase. The only tested compound that decreased FLU-R formation ex vivo was menadione, although there was an equivalent decrease in worm viability (viability and inhibition were tested in the same experiment); therefore, it is probable that the decreased amount of detected FLU-R was due to nematode toxicity rather than enzyme inhibition. Taken together, the results of our study show for the first time that all developmental stages of *H. contortus* can deactivate FLU via carbonyl group reduction. The ability to generate FLU-R increases as larvae develop, with adult IRE females having the highest CRE activity. Larvae and adult females of the drug-resistant IRE strain reduce FLU more effectively than those of the drug-susceptible ISE strain. Among the natural compounds tested with potential CRE-inhibiting properties, naringenin and menadione significantly inhibited the reduction of FLU in adult nematodes from both strains in vitro. Nevertheless, ex vivo, menadione was toxic to living nematodes, and naringenin did not inhibit the reduction of FLU. This toxicity of menadione might be advantageous if it not only improves FLU efficacy but also causes further loss of viability on its own. Given that menadione is already used in animal feed and is an approved biocide, it could potentially be easier to secure approval for its veterinary use than the other inhibitors. However, further studies are necessary.
